# The effect of tax incentives practices on the sustainability of micro, small and medium enterprises in Ethiopia during the outbreak of corona virus pandemic

**DOI:** 10.1186/s13731-022-00194-8

**Published:** 2022-01-28

**Authors:** Kanbiro Orkaido Deyganto

**Affiliations:** Department of Accounting and Finance, Dilla University, Dilla, Ethiopia

**Keywords:** Micro, Small and medium-sized enterprise, Tax incentives practices, Sustainability, Corona virus pandemic, Ethiopia

## Abstract

**Purpose:**

This study was aimed to identify the effect of tax incentive practices on the sustainability of MSMEs during an outbreak of coronavirus pandemic in Ethiopia.

**Design/methodology/approach:**

To achieve this objective, the researcher has employed a quantitative research approach with an explanatory research design in which six hypotheses have been tested. The primary data were collected from 300 MSMEs owners/operators using structured questionnaires. A multiple regression model was employed to study the effect of tax incentives parameters on the sustainability of MSMEs.

**Findings:**

According to the regression analysis, this paper revealed that tax holiday, tax allowance, reduction in the tax rate, accelerated depreciation, loss carry forward, and tax exemption have a positive and statistically significant effect on the sustainability of the MSMEs.

**Research limitations:**

The current study was geographically focused on Ethiopia by considering the MSMEs sector. The subject wise was focused on the effect of the tax incentives on the sustainability of MSMEs. The primary data were limited to the year 2020. It might be improved in the future if other researchers incorporate large firms in the country and use secondary data for the analysis of multiple years. Also, future researchers will improve the same study by considering both monetary and non-monetary incentives as study variables.

**Practical implications:**

To survive during the COVID-19 pandemic, MSMEs need support from the government in the form of tax incentives. The tax incentives play a great role in enhancing the growth and the sustainability of MSMEs as well as the economy as a whole. So the owners of MSMEs have to ask the government to obtain necessary support in the form of monetary and non-monetary incentives to be sustainable in economic activity. Also, the findings and recommendations of the current study might serve as an ingredient and be informative to the policymakers on the MSMEs sector. The governments have to continue to shape tax incentives policies to support the MSMEs’ sustainability by providing tax and non-tax incentives.

**Originality/value:**

This study contributes to empirical evidence about the effect of tax incentives practices on the sustainability of MSMEs during the outbreak of COVID-19 in Ethiopia by considering six tax incentive practices in terms of tax holiday, tax allowance, reduction in the tax rate, accelerated depreciation, loss carried forward, and tax exemption.

## Introduction

Promoting sustainable development goals (SDGs) are the top agenda of all governments in the world to achieve the living standard of their people. MSMEs are the key tool to achieve sustainable development goals. MSMEs are considered as an engine for the economy and used as key instruments for reducing poverty through promoting sustainable development goals (Jansson, et al., 2017). The MSMEs support the governments by reducing the unemployment rate and paying tax revenue to encourage economic development (Agyapong, 2010). To remain their contribution to economic growth, MSMEs have to be sustainable at any time (Tekola & Gidey, 2019). Even if MSMEs play a great role in world economic growth, MSMEs were hit by the outbreak of the coronavirus pandemic (Mogaji, 2020). The labor market impact of the COVID-19 pandemic reveals the devastating effect on workers in the informal economy and hundreds of millions of MSMEs worldwide, and as a result, MSME’s contribution to national GDP, creation of employment opportunity, and other benefits are being hampered. MSMEs are mostly at the bottom of the industrial chain and face problems of high energy consumption and low output in developing countries. Thus, studying the sustainability of MSMEs in emerging economies is necessary and solutions are needed to give them the support they need to survive and continue to contribute to the global economy (Engidaw, 2021).

Currently, the Government of Ethiopia has formulated 10-year strategic plans from the period 2020 to 2030 for becoming an African Beacon of Prosperity in 2030. Sustainable development goals can be achieved if MSMEs sustain themselves in the country. But, the pandemic led to massive damage to economic activities in general and MSMEs in particular following restrictions on human mobility by the government and stay-at-home advice by the ministry of health. These actions and advice, then, have adversely affected both the supply and demand side of MSME’s operation specifically, and the pandemic’s negative consequences on MSME’s include raw material supply was stopped, shortage of workers happened, shortage of working capital created, their operational cost increased, and decline in consumers consumption of products of MSMEs (Engidaw, 2021). So, the sustainability of MSMEs during COVID-19 pressure is a matter and burning issue of researchers and academicians across the world. It is time for the governments to support the MSMEs during this hard time to achieve the sustainability of MSMEs. The government can also take on new responsibilities to support wider government actions and help address the impact of the COVID-19 pandemic on their business activity. The tax incentive is the best government measure in place intended to encourage MSMEs by deduction, exclusion, exemption from tax liability for a certain period to sustain sustainability, and contribution to economic development. Tax incentives are ways of reducing taxes for businesses and encouraging those businesses and individuals to engage in behavior that is socially responsible and benefits the community. So that well-designed tax incentive practices have a positive and statistically significant effect on the growth and sustainability of MSMEs (Atawodi & Ojekal, 2012; Ahmedova, 2015; Twesige & Gasheja, 2019).

The topic effect of tax incentives practices on the sustainability of MSMEs in Ethiopia is overlooked and not researched well during the Outbreak of the Corona Virus Pandemic. To the level of knowledge of the researcher, few pieces of research have been conducted on a related topic in Ethiopia. For instance, Ayele (2006); Alene (2020); and Engidaw (2021) were addressed factors affecting micro and small business enterprises' performance in Ethiopia. The empirical studies ignored the effect of tax incentives practice on the sustainability of MSMEs during the COVID-19 pandemic because MSMEs in the country were hit by the pandemic. This is why the researcher was motivated to conduct this research work to fill the aforementioned research gaps. Specifically, the paper was designed to identify the effect of six tax incentive indicators, like tax holiday, tax allowance, decrease in the tax rate, accelerated depreciation, loss carry forward, and tax exemption, on the sustainability of MSMEs in Ethiopia during the COVID-19 pandemic. Hence, the findings and recommendations of the current study would serve as an ingredient and be informative to the government, MSMEs owners, and policymakers on the MSMEs sector.

The rest of the paper was organized as follows: section two reviews related to literature. Section three describes the methodology. Results and discussion is included in section four. Section five provides conclusions and recommendations. Lastly, section six provides direction for future research.

## Literature review

### Coronavirus disease

In early 2020, after a December 2019 outbreak in China, the World Health Organization identified SARS-CoV-2 as a new type of coronavirus. The outbreak quickly spread around the world. COVID-19 is a disease caused by SARS-CoV-2 that can trigger what doctors call a respiratory tract infection. It can affect your upper respiratory tract (sinuses, nose, and throat) or lower respiratory tract (windpipe and lungs). It spreads the same way other coronaviruses do, mainly through person-to-person contact. Infections range from mild to deadly. The World Health Organization (WHO) on March 11 declared COVID-19 a pandemic, pointing to the over 118,000 cases of coronavirus illness in over 110 countries and territories around the world and the sustained risk of further global spread. An epidemic refers to an uptick in the spread of a disease within a specific community. By contrast, the WHO defines a pandemic as the global spread of a new disease, though the specific threshold for meeting those criteria is fuzzy. The term is most often applied to new influenza strains, and the CDC says it is used when viruses “can infect people easily and spread from person to person in an efficient and sustained way” in multiple regions. The declaration refers to the spread of a disease, rather than the severity of the illness it causes (Ratten, 2020).

Coronavirus disease (COVID-19) is an infectious disease caused by a newly discovered coronavirus. Most people who fall sick with COVID-19 will experience mild to moderate symptoms and recover without special treatment. The virus that causes COVID-19 is mainly transmitted through droplets generated when an infected person coughs, sneezes, or exhales. These droplets are too heavy to hang in the air and quickly fall on floors or surfaces. You can be infected by breathing in the virus if you are within proximity of someone who has COVID-19, or by touching a contaminated surface and then your eyes, nose, or mouth. A coronavirus is a kind of common virus that causes an infection in your nose, sinuses, or upper throat. Most coronaviruses are not dangerous. Corona Virus emerged in Wuhan, China, spread across the world infecting more than 6.1 million and causing the death of more than 371,857 people as of June 1, 2020. In Africa, since the first case was reported in Egypt, the virus has spread to 53 countries within weeks. As of June 1, 2020, more than 146,996 cases and 4222 deaths were reported. In Ethiopia, a total of 1257 cases and 12 deaths were reported by the Ministry of Health on June 1, 2020(MOH, 2020) and Community Health Institute (2020). The outbreak of the COVID-19 pandemic has disturbed the political, economic, social, religious, and financial structure of the world (Mogaji, 2020).

### Definition of MSMEs

While there still lacks a universally accepted definition, MSMEs are widely recognized for the important contributions they make to sustainable development, in terms of contributions to economic growth, creation of decent jobs, provision of public goods and services, as well as poverty alleviation and reduced inequality. According to the revised MSMEs Growth Stages Guideline No. 004/2011, the revised definition considers used labor force, including family labor; total assets without working building and the division of sub-sector into service and industry are the main criteria (Table 1).

**Table 1:** Definition of MSMEs in Ethiopia

Level of enterprise	Sector	Employment	Total asset(in Birr)
Microenterprise	Industry sector: (includes manufacturing, construction, and mining sub-sectors)	A business enterprise which uses not over five labor force, including business owner	The monetary value of the enterprise’s total asset is not over 100,000 Ethiopian Br
	Service sector: (includes retail trade, transport, hotel, and tourism information technology and repairs)	A business enterprise which uses not over five labor force, including business owner	The total asset is not over 100,000 Ethiopian Br
Small enterprise	Industrial sector: (includes manufacturing, construction, and mining sub-sectors)	A business enterprise that uses 6–30 labor force, including business owners and family labor	The financial value of the enterprise’s total asset ranging from 100,001 to 1500,000 Br
	Service sector: (includes retail trade, transport, hotel, and tourism information technology and repairs)	A business enterprise that uses 6–30 labor force, including business owners and family labor	The monetary value of the enterprise’s total asset ranging from 50,001 to 500,000 Br
Medium-sized Enterprises	Industrial sector: (includes manufacturing, construction)	Above 30 labor	Birr 1,500,000 up to Birr 2 million
	Service sector: (includes retail trade, transport, hotel, and tourism information technology and repairs)	Above 30 labor	Birr 500,000 up to Birr 1 million

Source: Alene (2020)

Besides, the minister's proclamation number 201/2011 defines micro-enterprises as an enterprise having total capital of Br 50,000 not including building for service enterprise or not exceeding Br 100,000 for industrial enterprise. The proclamation also defines a small-scale enterprise as an enterprise having to total capital of Br 50,001 to 100,000 not including building and having 6–30 employees for service or having the capital of Br 100, 001 to 1,500,000 for the industry. Medium enterprises are these business enterprises with a total investment between Birr 500,000 up to Birr 1 million, including those enterprises that have high technical consultancy and excluding another high-tech establishment.

### Sustainability of MSMEs

Research shows that investing and enabling MSMEs to fulfill their development potential can have significant contributions to 60% of the SDG targets, including those related to goals SDG 8 (Promote sustained, inclusive, and sustainable economic growth, full and productive employment, and decent work for all) and SDG 9 (Build resilient infrastructure, promote inclusive and sustainable industrialization, and foster innovation), among others. Thus, investing in and supporting the sustainability of MSMEs in the world is an integral part of the sustainability efforts of clients, can yield significant progress towards an economy that works for all. MSMEs represent the lion’s share of businesses and employment, as they represent 99% of businesses and 67% of employment. Therefore, they are among the strongest drivers of economic development. However, despite their potential, MSMEs in the region tend to stay small and are significantly less productive than large firms (Inter-American Development Bank, 2021).

At IDB Invest we offer inclusive and sustainable financial solutions by supporting financial services clients to maximize their unique role in promoting sustainable and inclusive economic growth. We do this by integrating a sustainability lens in their investments, products, and services to their clients, and also by expanding our clients’ financial inclusion of MSMEs and underserved populations. MSMEs have a very important role in developing the Philippine economy. They help reduce poverty by creating jobs for the country's growing labor force. They stimulate economic development in rural and far-flung areas; MSMEs are one of the core engines of the Indian economy and are projected to grow further in the coming decade. MSMEs, especially manufacturing sector enterprises, are particularly resource-intensive and emit a high quantum of pollution and carbon emissions. MSMEs in Ethiopia are the chief sources of jobs and income, significantly contribute to the local, regional and national GDP and key policies to eliminate poverty (Tekola & Gidey, 2019).

### Empirical review of related studies

Emerging economies have introduced tax incentives for various reasons. In some countries in transition, such instruments may be seen as a counterweight to the investment disincentives inherent in the general tax system. In other countries, the incentives are intended to offset other disadvantages that investors may face, such as a lack of infrastructure, complicated and antiquated laws, bureaucratic complexities, and weak administration. In Ethiopia, with the increase in the number of people infected with COVID-19, the government declared a state of emergency aiming to curb the spread of the virus in the country. The state of emergency puts restrictions on travel and human mobility which hurt the economy and businesses particularly micro and small sectors. This effect requires more research-based evidence that shows the extent to which MSMEs are affected. Although reports are coming out from different media outlets, further evaluation is required to reveal the effect of the unprecedented pandemic on the MSMEs and provide possible solutions. So far no study is conducted on this issue. Now, it is time to respond to the mentioned gap through rapid assessment of the effect of Corona Virus on MSME’s operation and provide insight for decision-makers in the sector and researchers to conduct a further investigation (Lemi et al., 2020). Prior studies addressed the same issue and suggested that tax incentive indicators like tax holiday, tax allowance, decrease in the tax rate, accelerated depreciation, loss carryforward, and tax exemption on the sustainability of MSMEs.. The empirical findings are summarized as follows (Table 2):

**Table 2:** Summary of empirical review and hypotheses

Variable name	Empirical Studies findings
Tax holiday	Empirical studies, like Atawodi and Ojekal (2012); Tekola and Gidey (2019); Ahmedova (2015); Boso et al. (2017); Fernández-Viñé et al. (2013); Twesige and Gasheja (2019); and Jansson et al. (2017), were evidenced that tax holiday has positive effect On Sustainability Of MSMEs. So it can be hypothesized that H1: holiday has a positive and statistically significant effect on the sustainability of MSMEs
Tax allowance	Prior studies, such as Atawodi and Ojekal (2012); Ahmedova (2015); Boso et al. (2017); Fernández-Viñé et al. (2013); and Twesige and Gasheja (2019), were suggested that tax allowance has a positive effect On the Sustainability of MSMEs. So that a tentative statement could be developed H2: tax allowance has a positive and statistically significant effect on the sustainability of MSMEs
Reduction in tax rate	Studies were conducted by Ahmedova, (2015); Voronkova et al. (2018); Boso et al. (2017); Fernández-Viñé et al. (2013); and Twesige and Gasheja (2019) were reduction in tax rate has a positive effect on the sustainability of MSMEs. Hence, the hypothesis can be developed as follows: H3: reduction in tax rate has a positive and statistically significant effect on the sustainability of MSMEs
Accelerated depreciation	Experimental studies of Ahmedova (2015); Atawodi and Ojekal (2012); Boso et al. (2017); Fernández-Viñé et al. (2013); Twesige and Gasheja (2019); and Jansson et al. (2017) were evidenced that accelerated depreciation has a positive effect on the sustainability of MSMEs H4: accelerated depreciation has a positive and statistically significant effect on the sustainability of MSMEs
Loss carry forward	First-hand studies, like Ahmedova (2015); Boso et al. (2017); Fernández-Viñé et al. (2013); Twesige and Gasheja (2019); Jansson et al. (2017); and Atawodi and Ojekal (2012), were found out that Loss carry forward has a positive effect on the sustainability of MSMEs. So the researcher developed the tentative statement as follows: H5: Loss carry forward has a positive and statistically significant effect on the sustainability of MSMEs
Tax exemption	Previous studies were undertaken by Boso et al. (2017); Fernández-Viñé et al. (2013); Twesige and Gasheja (2019); Jansson et al. (2017); Atawodi and Ojekal (2012); and Ahmedova (2015) where evidenced tax exemption has a positive effect on the sustainability of MSMEs. It can be hypothesized that H6: Tax exemption has a positive and statistically significant effect on the sustainability of MSMEs

Source: own development based on the empirical review (2020)

### Conceptual framework

**Fig. 1:**
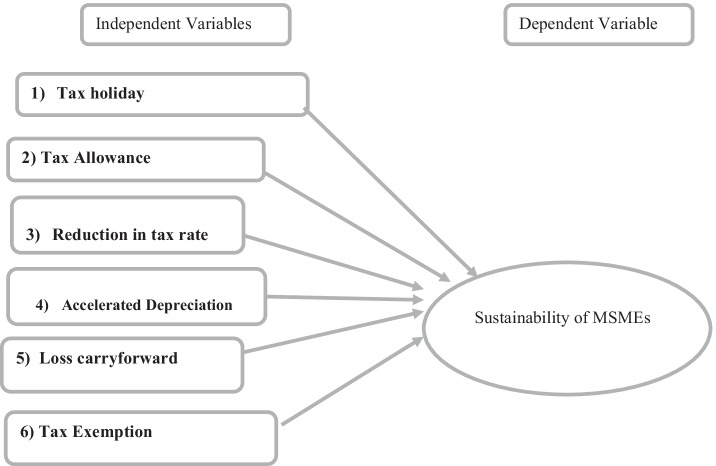
Conceptual Framework. Source: Own development based on literature review (2020)

The theoretical framework explains the entire research briefly; for this study, there are six explanatory variables, including tax holiday, tax allowance reduction in the tax rate, accelerated depreciation, loss carried forward, and tax exemption as explanatory variables, and the sustainability of MSMEs as the dependent variable (Fig. 1).

## Research methodology

### Research design and research approach

This paper has employed an explanatory research design because the objective of this study is to identify the effects of tax incentives on the growth of MSMEs in Ethiopia. The research approach that was employed in this study was a quantitative research approach due to the quantitative nature of the data.

### Target population, sampling techniques, and sample size determination

#### Target population

The target population was 2,490,645 MSMEs licensed operated in Ethiopia as of May 2020.

#### Sampling techniques

To select a sample size from 2,490,645 MSMEs, the researcher used a simple random sampling technique to give equal chances to MSMEs owners/operators.

#### Sample size determination

To determine the sample size for this study, the researcher has used the mathematical formula of Yamane (1967) by taking into account the total population, the sampling error, and the level of reliability, and it is assumed that the sample would have 95% reliability about population and a sampling error will be 5%. This simplest formula is.

$$n=\frac{N}{1+\left({0.05}^{2}\right)*N},$$

where *n* is the sample size; *N* is the target population; $\frac{\mathrm{2,490,645 }}{1+\left({0.05}^{2}\right)*\mathrm{2,490,645 }} =400$.

The four hundred sample size was distributed among Micro business Enterprises, Small-scale Enterprises, and Medium-Sized Enterprises in Ethiopia (Table 3).

**Table 3:** Sample size distribution

Sampling Unit	The population of each enterprise	Sample Size calculations	Sample size from each enterprise
Micro business Enterprises	930,215	(930,215/2,490,645) × 400	150
Small-scale Enterprises	830,215	(830,215/2,490,645) × 400	133
Medium-Sized Enterprises	730,215	730,215/2,490,645) × 400	117
Total	2,490,645	(2,490,645/2,490,645) × 400	400

Source: Federal Micro and Small Enterprises Development Agency (2020)

Therefore, the maximum sample size of this study was 400 MSMEs officers/owners in Ethiopia, which consists of 150 Microbusiness Enterprises, 133 Small-Scale Enterprises, and 117 Medium-Sized Enterprises.

### Operational definition of variables

#### Variables

The dependent variable is the sustainability of MSMEs and independent variables are six tax incentive indicators (tax holiday, tax allowance, decrease in the tax rate, accelerated depreciation, loss carry forward, and tax exemption). They have operationally defined as follows (Table 4):

**Table 4:** Summary of variables operational definition and scale of measurement

Variables incorporated in the model	Unit of measurement	Sign expected
Dependent variable		
Sustainability MSMEs: increasing in sustainability indicators, such as increasing number of employees, sales volumes, market share, customer base as highly decreased (1), decreased (2), constant (3), improved (4), and highly improved (5) since last 12 months	Likert scale questionnaire	
Explanatory variables		
Tax holiday: tax holidays are the temporary exemption of a new firm or investment from certain specified taxes, typically at least corporate income tax	Likert scale questionnaire	+
Tax allowance: tax allowance is a deduction of a certain fraction of investment from taxable profits (in addition to depreciation)	Likert scale questionnaire	+
Reduction in tax rate: reduction in tax rates leads to lower production, distribution, and selling costs which lead to higher prices, and as a result, consumers change their buying behavior	Likert scale questionnaire	+
Accelerated depreciation: accelerated depreciation is depreciation at a faster schedule than available for the rest of the economy. This can be implemented in different ways, including a higher first-year allowance, or increased depreciation rates. Total tax payments in nominal terms over time are unaffected, but their net present value is reduced and the liquidity of firms is improved	Likert scale questionnaire	+
Loss carry forward: loss carried forward refers to an accounting technique that applies the current year's net operating loss (NOL) to future years' net income to reduce tax liability. This results in lower taxable income in positive NOI years and reduces the amount the company owes the government in taxes	Likert scale questionnaire	+
Tax Exemption: a tax exemption is the right to exclude all or some income from taxation by federal or states governments. Most taxpayers are entitled to various exemptions to reduce their taxable income, and certain individuals and organizations are completely exempt from paying taxes	Likert scale questionnaire	+

Source: Own construct, 2020

### Econometric model specification

To examine the effect of tax incentives on the sustainability status of MSMEs, the multiple regression model has been employed to observe the relation of each tax incentive parameter with sustainability indicators, such as increasing number of employees, sales volumes, market share, customer base, and assets growth using Likert scale due to tax incentives provided to the enterprises for last 12 months during the existence of COVID-19. The equation of the model was expressed as:

$$\mathrm{Sustainability of MSME}= \beta 0+\left(\beta 1*\mathrm{Tax holiday}\right)+\left(\beta 2*\mathrm{Tax Allowance}\right)+\left(\beta 3*\mathrm{Reduction in Tax Rate}\right)+\left(\beta 4*\mathrm{Accelerated Depreciation}\right)+\left(\beta 5*\mathrm{Loss Carry Forward}\right)+\left(\beta 6*\mathrm{Tax exemption }\right)+U,$$

whereas *Ü* = error term.

### Data collection instruments and methods analysis

To collect appropriate and sufficient data for the study structured questionnaire were used. It has prepared both in English and Amharic languages to minimize language understanding barriers. This has been done because most MSMEs were not understood the English language. After the accomplishment of the data collection procedure, it was classified as per each variable; the qualitative data were coded to be measured quantitatively. In this research, both descriptive and inferential statistics will make with the help of SPSS version 21.0.

### Reliability and validity test

#### Reliability test

To test reliability, the researcher employed Cronbach’s Alpha (α) which is the most common measure of reliability and a value greater than 0.7 is very acceptable. This has been tested as follows (Table 5):

**Table 5:** Reliability Statistics

Cronbach’s Alpha	No. of items
0.947	44

Source: Survey data and SPSS result, 2020

Result depicted that the value Cronbach's Alpha value for the whole 44 items is valid for 0. 947, which means that the instrument has a high level of consistency (above 0.85). This indicates that all the variables under consideration accounts above the scientifically accepted threshold; therefore, the study is reliable under this circumstance. Compared with the minimum value of alpha 0.70 advocated by Cronbach’s (1951), then the responses generated for all of the variables used in this research were reliable enough for data analysis. This implies that the data incorporated in SPSS is reliable.

#### Validity test

Test validity is the extent to which a test accurately measures what it is supposed to measure. In this research, the researcher employed exploratory factor analysis to test the validity of the questionnaire. Before running exploratory factor analysis, KMO and Bartlett's tests have been carried out.

### KMO and Bartlett's test

Table 6 shows two tests that indicate the suitability of your data for structure detection. The Kaiser–Meyer–Olkin Measure of Sampling Adequacy is a statistic that indicates the proportion of variance in your variables that might be caused by underlying factors. High values (close to 1.0) generally indicate that factor analysis may be useful with your data. If the value is less than 0.50, the results of the factor analysis probably will not be very useful. Bartlett's test of sphericity tests the hypothesis that your correlation matrix is an identity matrix, which would indicate that your variables are unrelated and therefore unsuitable for structure detection. Small values (less than 0.05) of the significance level indicate that factor analysis may be useful with your data. This test specifies factor analysis is suitable for testing validity.

**Table 6:** KMO and Bartlett’s test

Kaiser–Meyer–Olkin measure of sampling adequacy	0.889
Bartlett's test of sphericity Approx, Chi-square	8230.901
* df*	136
Sig	0.000

Survey data and SPSS result, 2020

Table 7 presents the results of Exploratory Factor Analysis (EFA) carried out on the current study and the results were obtained from SPSS. The items with less than 0.5 associated variable load factor will be deleted, but there is no item with less than 0.5 load factor. The value of all items is greater than five of the items, which is kept to examine the variables. This showed that the validity is satisfied through Exploratory Factor Analysis.

**Table 7:** Exploratory factor analysis

Items	1	2	3	4	5	6	7
MSMEs Sustainability							
There is an increase in profitability compared to main competitors during the last 12 months	0.865						
There is an increase in sales volumes compared to main competitors during the last 12 months	0.819						
There is an increase in Market share compared to similar business during the last 12 months	0.540						
There is an increased Customer base compared to main competitors during the last 12 months	0.661						
There is an increasing number of employees compared to main competitors during the last 12 months	0.570						
There is increased Assets growth compared to main competitors since the last 12 months	0.843						
There is increased Product quality compared to main competitors during the last 12 months	0.810						
There is increase Production levels compared to main competitors during the last 12 months	0.570						
There is increase The overall performance compared to main competitors during the last 12 months	0.848						
Tax holiday							
Tax holiday incentives existing in the country is too costly to become beneficiary for MSMEs		0.830					
Government make publicly available information about tax holiday incentives for MSMEs		0.726					
The country adopted an equivalent standard for granting tax holiday incentives for MSMEs		0.861					
Everyone of MSMEs knows that which authority has the final decision on whether to grant tax holiday incentives to the specific investment		0.861					
MSMEs operators know that where does the country’s tax incentives regime provisions include tax holidays		0.814					
The tax holiday incentive is known and in practice in your enterprise		0.504					
Our enterprise has given the tax relief due to purchasing business equipment		0.731					
Tax allowance incentives existing in the country is too costly to become beneficiary for MSMEs		0.549					
Tax allowance							
State make publicly available information about tax allowance incentives for MSMEs			0.710				
The country adopted an equivalent standard for granting tax allowance incentives for MSMEs			0.640				
MSMEs knows that which authority has the final decision on whether to grant tax allowance incentive to the specific investment			0.841				
Operators of MSMEs know that where the country’s tax incentives regime provisions include tax allowance			0.740				
The tax allowance incentive is known and in practice in your enterprise			0.641				
Accelerated depreciation							
Accelerated depreciation provides MSMEs a way of deferring income taxes by reducing taxable income in current years				0.856			
I believe that accelerated depreciation is a valuable tax incentive that encourages SMEs to purchase new assets				0.707			
Our enterprise uses the double-declining balance method to calculate the depreciation expense of fixed assets by SMEs				0.683			
Our firm has always used accelerated depreciation during the last five years				0.571			
Accelerated depreciation is expected to be much more productive during its early years				0.480			
The government of Ethiopia charged a lower tax rate for encouraging MSME				0.824			
I believe that the tax rate of SMEs is lower in completion sole proprietorship and partnership				0.825			
Reduced tax rate							
Deduction of tax rates for SMEs is helpful to increase retained profits for reinvestment purposes					0.971		
The government protects the market for MSMEs import duties and promoting of purchases of locally produced goods by reducing the tax rate					0.514		
When reduced tax incentive plans are in place, employees recognize that significant effort on their behalf will be acknowledged and rewarded					0.848		
The tax authority of Ethiopia charged a lower tax rate for encouraging MSME					0.830		
I believe that the tax rate of MSMEs is lower than other business enterprises					0.726		
loss carried forward:							
The government of Ethiopia charged a lower tax rate for encouraging MSME						0.947	
Our enterprise granted Loss carry forward by the government						0.948	
A loss carry forward refers to an accounting technique that applies the current year's net operating loss (NOL) to future years' net income to reduce tax liability						0.944	
A tax loss carry forward allows taxpayers to use a taxable loss in the current period and apply it to a future tax period						0.948	
Government Provides to use a tax loss carry forward for several different purposes						0.944	
The loss of income in the current year cannot be carried forward if an ITR reporting the loss has not been filed within the due date						0.947	
Tax exemptions							
A tax exemption is the right to exclude all or some income from taxation by federal or states governments							0.947
I believe that tax exemption boosts our business sustainability							0.948
Tax exemption MSMEs is helpful to increase retained profits for reinvestment purposes							0.947
Tax incentives can have both positive and negative impacts on an economy							0.948

Survey data and SPSS result, 2020

### Ethical considerations

The ethical considerations given attention by the researchers and enumerators while conducting the research that includes voluntary participation, no harm would to participants, anonymity, and confidentiality, not deceiving the subjects, and privacy of participants. The finding of this study will encourage the sustainability of MSMEs other than harming them.

## Results and discussion

### Response rate

This part deals with the analysis and discussion of data collected from 300 respondents out of 400 MSMEs officers/owners in Ethiopia. The response rate was 75% consists which imply almost more than 50% of MSMEs owners/operators have been taking part in the process of data collection. Then, the analysis of the data was based on the availability of tax incentives offered to MSMEs, descriptive statistics, person correlation matrix, linear regression model assumptions, regression analysis, and hypotheses testing.

### Availability of tax incentives offered to MSMEs

Before collecting data in the form of a five-point Likert scale, the researcher asked the owners/operators of MSMEs whether they offered any kind of tax incentives by tax authority during an outbreak of COVID-19 to check the existence of tax incentive practices in Ethiopia (Table 8).

**Table 8:** Tax Incentives offered to MSMEs

Do you ever offer tax incentives by tax authority during an outbreak of COVID-19?		Frequency	Percentage
Valid	No	0	0
	Yes	300	300
	Total	300	100.0

Source: Personal survey, 2020

The role of tax incentives in enhancing the sustainability of micro, small, and medium-sized enterprises is a very significant issue because the sustainability of small and medium-sized enterprises promotes the economic development of the nation in general. In this study, more than 100% (300) used tax incentives provided by the tax authority. If other things are constant, the most MSMEs in the city will achieve their sustainability.

### Descriptive statistics results

Descriptive statistics are very important because if we simply presented our raw data it would be hard to visualize what the data were showing, especially if there was a lot of it. Descriptive statistics, therefore, enables us to present the data in a more meaningful way, which allows a simpler interpretation of the data. In this study, descriptive statistics were analyzed as follows:

The Likert scale questionnaire of sustainability has 5 maximum and 1 minimum values respectively. The standard deviation value of 1.20246 was indicated that there was a variation of actual responses from the mean. About other variables the tax holiday 2.6000 with (SD) 1.11541, tax allowance of mean 3.1000, (SD) of 1.32509, reduction in tax rate 3.2804 with SD of 0.96692, accelerated depreciation 2.9980 with SD 0.75470, loss carry forward 0.8300 with SD of 1.24348 and tax exemption with 2.8700 with SD 0.96759 have the overall mean and standard deviation, respectively. In short, all variables incorporated in the model have a moderate contribution to the response variable sustainability of MSMEs. But the mean value of the variables could not be considered for interpretation since it was affected by the extraneous values.

### Correlation analysis

Correlation analysis measures the relationship between two items. The correlation matrix for this study was computed as follows:

With regard to the relationship between the sustainability of MSMEs and independent variables with a coefficient of correlation 1 indicates that each variable is perfectly correlated with each other. The result of the correlation analysis in Table 9 shows that tax holiday, tax allowance, reduction in the tax rate, accelerated depreciation, loss carried forward, and tax exemption were positive and significantly correlated with sustainability at a 1% level of significance. The correlation among independent variables is not more than 0.8. This implies that there is no multicollinearity problem in the model and the values of correlation are reliable.

**Table 9:** Summary of descriptive statistics

Variables	*N*	Minimum	Maximum	Mean	Std. deviation
Sustainability of MSMEs	300	1.00	5.00	2.6700	1.20246
Tax holiday	300	1.00	5.00	2.6000	1.11541
Tax allowance	300	1.00	5.00	3.1000	1.32509
Reduction in tax rate	300	1.00	5.00	3.2804	0.96692
Accelerated depreciation	300	1.00	5.00	2.9980	0.75470
Loss carry forward	300	1.00	5.00	2.8300	1.24348
Tax exemption	300	1.00	5.00	2.8700	0.96759

Sources: Survey data, 2020

### Assessment of ordinary least square assumptions

The most common assumptions to be tested before running the final regression result are normality, multicollinearity, autocorrelation, and heteroscedasticity.

### Assumption #1: the values of the residuals are normally distributed (normality test)

The Classical Linear Regression Model assumes that the error term is normally distributed with the mean of error being zero as the positive error will offset the negative error. This assumption can be tested by looking at the distribution of residuals. We can do this by checking the histogram this has shown as follows:
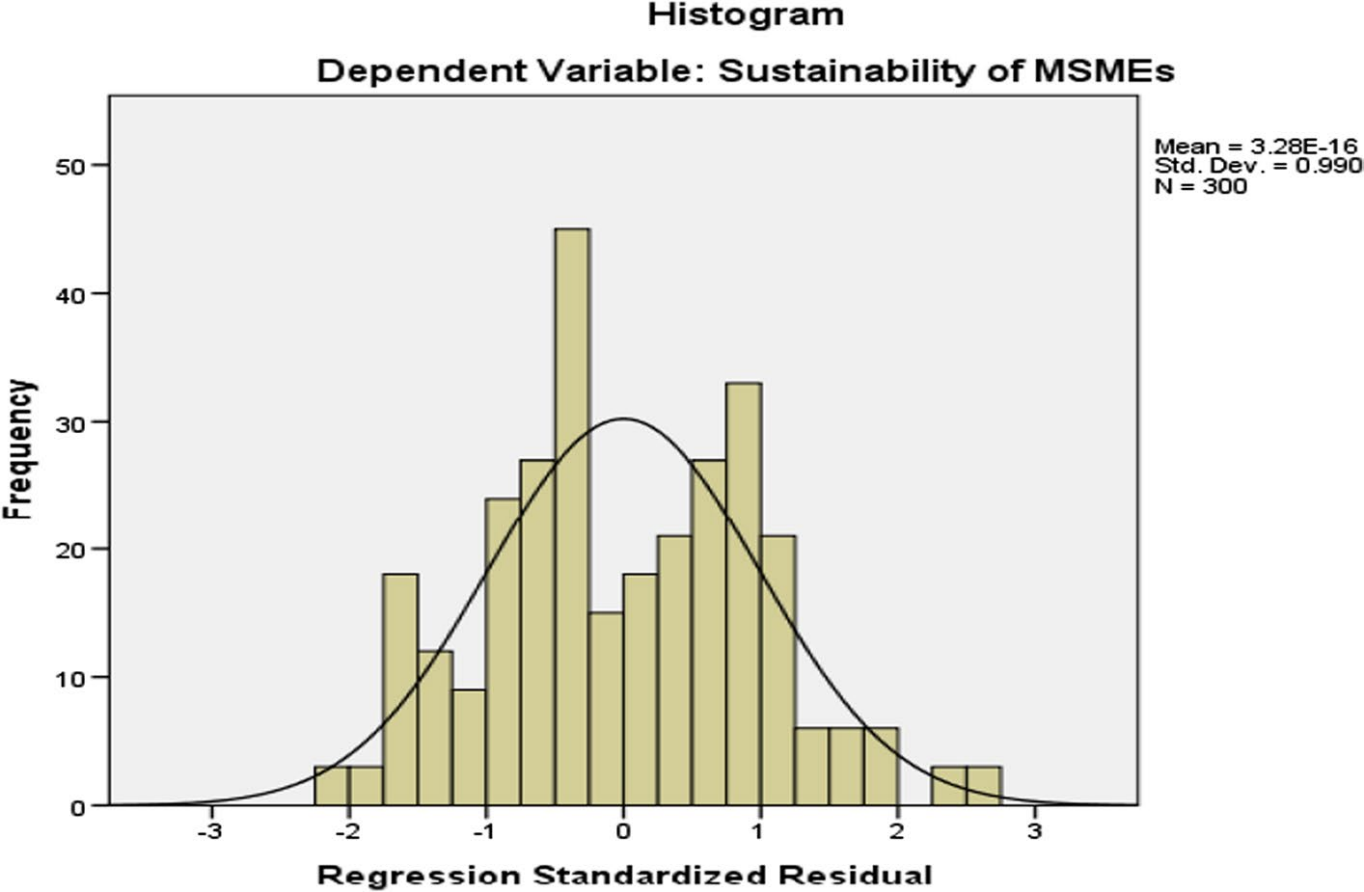


Source: Personal Survey data, 2020.

Based on the results shown above, the histogram on the distribution of residuals which is bell shaped is linear to the regression line from the SPSS output. So, the researcher concluded that there is no normality problem (the values of the residuals are normally distributed**)** on the data used for this study (Table 10).

**Table 10:** Pearson correlation matrix of the variables

Variables	1	2	3	4	5	6	7
Sustainability of MSMEs (1)	1						
Tax holiday (2)	0.350^**^	1					
Tax allowance (3)	0.153^**^	0.285^**^	1				
Reduction in tax rate (4)	0.257^**^	0.132^*^	0.060	1			
Accelerated depreciation (5)	0.067**	0.064	− 0.181^**^	0.264^**^	1		
Loss carry forward (6)	0.352^**^	− 0.129^*^	− 0.452^**^	0.045	0.005	1	
Tax exemption (7)	0.368^**^	− 0.262^**^	− 0.021	0.017	− 0.184^**^	0.399^**^	1

**Correlation is significant at the 0.01 level (2 tailed)

*Correlation is significant at the 0.05 level (2 tailed)

Source: Survey data, 2020

### Assumption #2: test for multicollinearity

This is essentially the assumption that your predictors are not too highly correlated with one another. The tolerance levels for all variables are greater than 0.10 and the VIF value is less than 10; then we can conclude that predictors are not too highly correlated with one another.

As shown in the collinearity Table 11, the tolerance levels for all variables are greater than 0.10 and the VIF value is less than 10. This indicates that there were no multicollinearity problems that alter the analysis of the findings; rather it leads to the acceptance of R-value, tolerance, and VIF values.

**Table 11:** Collinearity statistics

Variables	Collinearity statistics	
	Tolerance	VIV
Tax holiday	0.824	1.214
Tax allowance	0.658	1.519
Reduction in tax rate	0.897	1.115
Accelerated Dep	0.852	1.174
Loss carry forward	0.629	1.590
Tax exemption	0.720	1.388

Source: Personal survey data, 2020

### Assumption #3: the values of the residuals are independent (autocorrelation)

This is the same as saying that we need our observations (or individual data points) to be independent of one another (or uncorrelated). We can test this assumption using the Durbin-Watson statistic, through SPSS. The Durbin-Watson closer to 2 or more is acceptable. The Durbin-Watson statistics value which is close to 2, equal to 2, or more suggests that there is no autocorrelation among error terms. Accordingly, the Durbin-Watson statistics value of 1.932 is close to 2, which indicates that autocorrelation is not a threat to the use of OLS in this study (see regression Table 7). Therefore, it can be concluded that the values of the residuals are independent which implies the absence of a serial correlation problem in our regression analysis.

### Assumption #4: the variance of the residuals is constant (heteroscedasticity test)

**Fig. 2:**
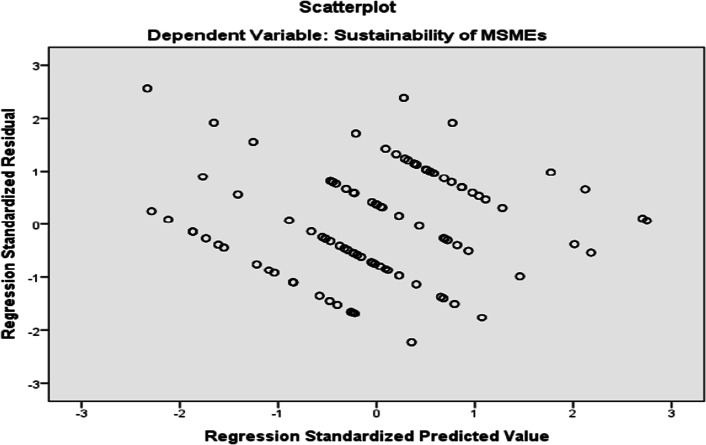
Assessment of heteroscedasticity using Scatter plot. Source: Personal survey data, 2020

This is called homoscedasticity and is the assumption that the variation in the residuals (or amount of error in the model) is similar at each point across the model. In other words, the spread of the residuals should be fairly constant at each point of the predictor variables (or across the linear model). We can get an idea of this by looking at our original scatterplot but to properly test this, we need to ask SPSS to produce a special scatterplot for us that includes the whole model (and not just the individual predictors). To test the 4th assumption, we need to plot the standardized values our model would predict, against the standardized residuals obtained. This has shown as follows (Fig. 2):

The above figure showed that there is homoscedasticity and the assumption that the variation in the residuals (or amount of error in the model) is similar at each point across the model is satisfied. So, we can conclude that there is no heteroscedasticity problem in the model.

### The regression analysis (inferential statistics): sustainability of MSMEs

As long as the model satisfies the OLS assumptions for linear regression, the regression coefficients give us the best possible estimates. Before running the final regression analysis, this study tested normality, multicollinearity, autocorrelation, and heteroscedasticity assumptions and confirmed that all the assumptions were satisfied in the aforementioned section. Then, the regression analysis was done as follows.

The multiple linear regression results are presented in Table 12; the adjusted R-squared statistics of the model was 46.1 percent. The result indicates that 46.1 percent variation in the dependent variable was jointly explained by the explanatory variables in the model, whereas the remaining 53.9 percent of the variation in the sustainability of MSMEs (as measured by the Likert scale) is explained by other variables which are not included in the model. The coefficient of explanatory variables tax holiday 0.433, tax allowance 0.199, reduction in tax rate 0.178, accelerated deprecation 0.170, loss carry forward 0.341, tax exemption 0.441 implies that 1% increase in the variables leads to 43.3%,19.9%, 17.8%, 17%, 34.1%, and 44.1% increase in sustainability of MSMEs. Besides, the F-statistics (43.583) in the model summary and ANOVA with (p-value of 0.000) were used to test the overall significance of the model and indicated the reliability and validity of the model at a 1% level of significance. This tells us that the model as a whole is statistically significant.

**Table 12:** Regression results

*R* = 0.687, *R*^2^ = 0.472, Ad *R*^2^.461,Std. Error of the Estimate = 0.88299, Durbin-Watson (*d*) = 1.932, *F*-statistic = 43.583, *p*-value = 0.000, ANOVA with (*p*-value of 0.000										
Model		Unstandardized Coefficients		Standardized Coefficients	*T*	Sig	95.0% Confidence Interval for B		Collinearity Statistics	
		*B*	Std. Error	Beta			Lower Bound	Upper Bound	Tolerance	VIF
	(Constant)	− 2.378	0.378		− 6.285	0.000	− 3.123	− 1.633		
	Tax holiday	0.433	0.050	0.401	8.578	0.000**	0.333	0.532	0.824	1.214
	Tax Allowance	0.199	0.047	0.217	4.139	0.000**	0.103	0.290	0.658	1.519
	Reduction in Tax Rate	0.178	0.056	0.141	3.136	0.002**	0.065	0.285	0.897	1.115
	Accelerated Dep	0.170	0.073	0.107	2.314	0.021*	0.025	0.314	0.852	1.174
	Loss Carry Forward	0.341	0.052	0.353	6.594	0.000**	0.240	0.443	0.629	1.590
	Tax exemption	0.441	0.062	0.355	7.088	0.000**	0.318	0.563	0.720	1.388

The dependent variable, sustainability of MSMEs, **Correlation is significant at the 0.01 level, *Correlation is significant at the 0.05 level (2 tailed)

Source: Personal Survey data, 2020

## Discussion

The result of this study shows that tax holiday with a coefficient of regression [β = *0*.433] has a positive and statistically significant effect on the sustainability of MSMEs at a 1% level of significance since (*p*-value of 0.000 > 0.01). Hence, hypothesis one is accepted. This finding is consistent with the finding of other studies results, such as Atawodi and Ojekal (2012); Tekola and Gidey (2019); Ahmedova (2015); Boso et al. (2017); Fernández-Viñé et al. (2013); Twesige and Gasheja (2019); and Jansson et al. (2017) who evidenced tax holiday positive effect on the sustainability of MSMEs in Ethiopia. This implies that the existence of a tax holiday enhances the sustainability of MSMEs operating in Ethiopia. Secondly, the coefficient of regression tax allowance [β = 0.199] is positive and statistically significant at a 1% level of significance with (p-value of 0.000 > 1%). Therefore, hypothesis two stated is accepted. This finding is consistent with the general logic that the existence of tax freedom enables to accumulation of capital since there are no annual payments to the government. It is also consistent with empirical findings of Atawodi and Ojekal (2012); Ahmedova (2015); Fernández-Viñé et al. (2013); and Twesige and Gasheja (2019) which suggested that tax allowance has a positive effect on the sustainability of MSMEs. This evidenced that tax allowance has a positive and significant effect on the sustainability of MSMEs.

The result of coefficient of [β = 0.178] is positive and statistically significant at a 1% level of significance because the sig of 0.002 is less than 1%. Hence, hypothesis three is accepted. This finding is consistent with the empirical findings of Atawodi and Ojekal (2012); Ahmedova (2015); Fernández-Viñé et al., (2013); and Twesige and Gasheja (2019) where reduction in tax rate has a positive effect on the sustainability of MSMEs. This tells us tax rate reduction has higher contribution tax sustainability of MSMEs. On the other hand, the accelerated depreciation variable has a coefficient of regression is [β = 0.170] is positive and statistically significant with (*p*-value of 0.021) which is significant at a 5%level of significances. Therefore, hypothesis four is accepted. This finding is consistent with regression results of studies by Boso et al. (2017); Fernández-Viñé et al. (2013); and Twesige and Gasheja (2019) which evidenced that accelerated depreciation has a positive effect on the sustainability of MSMEs. This indicates that considering the higher amount of accelerated depreciation amount from income tax has a positive effect on the sustainability of MSMEs in Ethiopia.

Concerning the loss carry forward, the result of this study shows that the increase in loss carried forward with a coefficient of regression [β = 0.341] has positive and statistically significant at 5% level of significance (*p* value of 0. 0.00 < 1% level of significances). Hence, hypothesis five is accepted. The result is similar with findings of Ahmedova (2015); Boso et al. (2017); Fernández-Viñé et al. (2013); Twesige and Gasheja (2019); and Twesige and Gasheja (2019) that found out that loss carry forward has a positive effect on the sustainability of MSMEs. It can be concluded that loss carry forward has a positive and significant effect on the sustainability of MSMEs. Last but not least, a tax exemption with a coefficient of regression of [β = 0.441] has a positive and significant effect on the sustainability of MSMEs with a *p* value 0.000 < 5% level of significance. Therefore, hypothesis six is accepted by the researcher. This finding is consistent with the empirical result of Fernández-Viñé et al. (2013); Twesige and Gasheja (2019); Atawodi and Ojekal (2012); and Ahmedova (2015) where evidenced tax exemption has a positive effect on the sustainability of MSMEs. This implies that tax exemption for MSMEs has a positive contribution to the sustainability of MSMEs.

## Conclusion and recommendations

In this paper, the researcher explored the effect of tax incentive parameters, such as tax holiday, tax allowance, tax reduction, accelerated depreciation, and loss carry forward, on the sustainability of MSMEs. By keeping this objective in mind, the researcher collected the primary data through a self-administrated questionnaire and analyzed it through SPSS version 21.0. A multiple regression model was employed to test the hypothesis. The conclusion that can be drawn from the findings was that hypotheses one up to six have been accepted by the researcher. This tells us an increase in the tax holiday, tax allowance, reduction in the tax rate, accelerated depreciation, loss carry forward, and tax exemption, which lead to an increase in business sustainability of MSMEs. This means the existence of tax incentive practices promotes the existence of MSMEs. Hence, to attract MSMEs expansion sustainability, governments should give top priority to improving their countries’ MSMEs business climates by adopting additional tax incentive practices to reduce the negative effect of the COVID-19 pandemic on overall business to improve and promote the economic development of the nation while minimizing the diverse effect of coronavirus on the survival and growth of MSMEs. The policymakers should continue to shape tax incentives policies by taking into account the parameters, like tax holiday, tax allowance, reduction in the tax rate, accelerated depreciation, loss carry forward, and tax exemption, to support the sustainability and growth of MSMEs across the world. Besides, the MSMEs business owners/operators have to understand and work on appropriate implementation of tax and non-tax incentives provided for them to sustain their business performance and contribution to economic development.

### Further research direction for improving limitations of this study

No study is free of limitations; accordingly, there are limitations in the current study that needs future improvements. This study was geographically focused on Ethiopia by considering the MSMEs sector. The subject wise was focused on the effect of the tax incentives (tax holiday, tax allowance, accelerated depreciation, reduction in the tax rate, loss carried forward, and tax exemptions) and on the sustainability of MSMEs during the existence of COVID-19. It was set out to investigate the effect of six tax incentives indicators. This study used only primary data which are limited to the year 2020. Hence, this study can be enhanced/improved if it is done at continental and global levels and also it is possible if it is done by using different methodology and sampling techniques. Further researchers can also study the topic of non-tax incentives together with the sustainability of MSMEs since the current variables explained about 46.1% and the remaining 53.9% variation on the growth of MSEs that were not incorporated in the current study. The impact of the COVID-19 pandemic on the overall performance of MSMEs should be further investigated with a combination of monetary incentives and non-monetary incentives to be more confident in this study.

## Authors' information

Mr. Kanbiro Orkaido Deyganto has earned the MBA in Finance in 2017 G.C. Currently, he was working at Dilla University as a lecturer and researcher in the Department of Accounting and Finance. He has been published about 20 articles in areas of accounting, auditing, small business enterprise performance, capital structure, public finance and taxation, and credit risk management on different reputable journals with 53 citations counted on the google scholar website.

## Data Availability

The data are included in this manuscript.

## Abbreviations

COVID-19: Coronavirus disease
MSMEs: Micro, small and medium-sized enterprises
IDB: Inter-American Development Bank
SDGs: Sustainable development goals
